# English Translation Ability for Cultivation Mode Based on Multimodal Auxiliary Scaffolding Teaching Method

**DOI:** 10.1155/2022/3556703

**Published:** 2022-07-19

**Authors:** Jie Meng

**Affiliations:** ^1^College of Applied Engineering, Henan University of Science and Technology, Sanmenxia 472000, Henan, China; ^2^Sanmenxia Polytechnic, Sanmenxia 472000, Henan, China

## Abstract

English learning occupies a very important position in college students' learning, and English translating is a skill that must be mastered to learn English, and translating can be used to measure the comprehensive level of college students' English learning. However, surveys show that English translation is still a difficult part of the English learning process for college students. Traditional translation teaching only focuses on the result and ignores the process; the classroom atmosphere is dull; students are not highly motivated to write; and students' knowledge of translating and its skills are lacking. Therefore, translating teaching should combine results and process and explore teaching methods that can improve students' interest and translating ability. In this paper, scaffolded teaching is the main teaching method, and multimodal teaching is used to support scaffolded teaching in college English translating teaching. This study provides experimental support for the interactive group teaching method. It also provides a feasible teaching method option for college English teachers to implement student-centered teaching practices and helps college English teachers transition from the traditional teacher-oriented indoctrination classroom teaching method to a student-centered teaching method that combines English skill development and skill application. In addition, students in the experimental group were equipped with the ability to self-correct and spontaneously improve on problems that occurred in their language. This study finds that in the group interactive cooperative teaching method, the teacher's heuristic teaching with questions to promote answers can effectively improve college students' intrinsic motivation to learn English; students' cooperative exploration of the questions raised by the teacher in the group is conducive to cultivating students' autonomy in English learning; in the teaching activities, the use of teaching materials to train students' skills instead of sentence-by-sentence translation and grammatical analysis of sentence structures can help students master At the same time, the interactive and cooperative group teaching method, which involves students in learning through group activities, can effectively improve students' English performance.

## 1. Introduction

As internationalization accelerates, the demand for people who can communicate proficiently in English has increased in more and more industries [1]. Due to the widespread use of English, people from different countries tend to use it more often as a tool for communicating with each other. English is becoming more and more important in the communication between home and abroad [2]. There is no great independent learning motivation for the improvement of English listening and speaking ability. The speaking test is not a required subject in the Level 4 and 6 exams for college students; therefore, even college students who pass the Level 4 and 6 exams with high scores do not reach the level of the Common European Framework for Language Teaching and Assessment (i.e., reach proficient communication in daily working life) [3]. Despite having studied English for more than ten years, from third grade to university and even in the workplace, they are still afraid to speak when confronted with foreigners and believe that their English skills are not good enough. The author's survey on teaching English to non-native students (including those from Asian countries) during their studies in the UK found that the number of hours of study to reach B2 level in spoken English should be 500–600 hours [4].

Through the actual investigation of “teaching” and “learning” in college English classroom, I found that although the “student-centered” teaching mode has been strongly advocated and publicized, many colleges and universities' I found that although the “student-centered” teaching mode has been strongly advocated and promoted, the English class mode in many colleges and universities is still what we call “teacher-centered” indoctrination education. In this widely used teaching style, the teacher is the core of the classroom and dominates the whole teaching activity as a knowledge authority, knowledge giver, and error corrector [5]. Through classroom observation records, the author found that English classes in colleges and universities are characterized by dogmatization and homogenization [6]. For example, in one of the classes observed by the author, the teacher first explained new words and introduced relevant background information, then translated and analyzed the text word by word and explained grammar; students were passively listening and taking notes [7]. The teacher did not give students the opportunity to practice speaking and daily communication in the classroom. This teaching mode belongs to the teacher-centered indoctrination teaching mode, which focuses more on the mechanical memorization of words and grammatical structures and the translation practice and analysis of texts. Students trained in this way can achieve a good level of English reading and translating, but have difficulty understanding actual conversations that take place in real situations and appear afraid to speak English [8]. The students are characterized by a scale of “deaf” English. Secondly, because the explanation of each text is focused on word-by-word translation and analysis, students lack learning skills such as skimming and sweeping, which are necessary for reading [9]. The traditional English translating teaching atmosphere is boring, and English as a foreign language remains difficult for students to master [10]. A review of the literature reveals that the scaffolding model has been applied in multiple disciplines and has proven to be an effective teaching method [11].

This paper analyzes the multimodal theory and scaffolding theory, and proposes a new teaching mode with specific application to the English translating classroom by combining the current situation, characteristics and objectives of English translating teaching in college, and exploring its application effects to find a strategy applicable to English translating teaching in college, aiming to change the current situation of English translating teaching and improve the English translating ability of students. The two classes with the same number of students and comparable grades and usually taught by the same teacher were selected as the experimental class and the control class through a pretest, and the multimodal assisted scaffolding teaching model was applied to the experimental class, while the control class was still taught by the traditional method and other experimental conditions were the same. After the experiment, the two classes were tested separately and the performance data were analyzed by SPSS. Based on the analysis results, the effectiveness of the new model in teaching was discussed, and reflections and corrections were made based on the results to finally improve the application of the model in the field of education. The results showed that after multimodal scaffolded translating instruction, students' interest and enthusiasm in translating learning improved, and students' daily translating habits also improved. The results showed that the students' interest and motivation in translating learning increased and their daily translating habits improved.

## 2. Related Work

According to the EC Distance Education and Training Project, scaffolding is defined as: scaffolding should provide a conceptual framework for the construction of the learner's understanding of knowledge [12]. In order to help students access their most recent developmental zone, scaffolding provides various forms of support for different levels of learners in a given setting; giving appropriate scaffolding to learners who are unable to perform tasks independently, helping them to better understand the information until the students independently use the new skill or strategy, and then the teacher gradually removes the scaffolding with the goal of developing students' learning abilities and helping them construct knowledge. Scaffolding as a teaching theory helps teachers divide the teaching process into five parts: (1) building a scaffolding platform and establishing a framework for learning thinking based on the “zone of most recent development” theory, (2) entering the situation, teachers lead students into the virtual simulation according to the scaffolding model according to the needs of the curriculum, (3) exploring independently and guiding students into the virtual independent exploration, (4) collaborative learning, negotiating, and discussing in small groups, and (5) evaluating learning ability, learning performance, and group activities [13].

Constructivism holds that people's life experiences are the most fundamental source of knowledge, and that the generation and construction of knowledge is accomplished through the summation of life experiences [14]. Constructivism developed its theory based on philosophical theory, which became a theoretical basis for the scaffolded teaching model in its subsequent development [15]. Constructivist theory can be seen in many fields, and Hartman argues that it contains two thoughts: social constructivism and cognitive constructivism. In students' learning process, they construct new ideas or concepts that depend on previous and current knowledge they possess. First, learners formulate hypotheses, select and substitute information, and then make decisions based on a cognitive structure [16]. Constructivism, with its focus on self-construction, is more concerned with the social meaning of the learner, and from what has been learned earlier, knowledge is obtained by summarizing the construction of people's life experiences, which was first proposed by the Swiss psychologist Jean Piaget. Later, many constructivist colleges appeared in different fields, while the focus was always on the constructive communication between learners and others and their communicative environment [17]. This view reveals that knowledge is constantly changing in the process of constructing human society and views learning as a process of personal understanding and constructing knowledge. From the viewpoint of the learners' memory system, they search for information, then transform and reorganize the extracted information, understand the new information through their previous knowledge, and finally, there is the most important part: completing the updating of the memory system and constructing a complete knowledge system based on the knowledge in the memory system [18].

The construction of knowledge can also take place through mutual exchange between people, each of whom is an independent individual with different personal experiences [19]. The nearest developmental zone is not in flux; it is a dynamic goal that develops further as students' abilities improve. Therefore, teachers should create a scaffold that is slightly higher than the students' current abilities in order to stimulate their interest in learning and to stimulate their latent abilities [20]. The physical medium, which is a long-standing source of forming social meanings, can convey the desired meaning through different societies, and thus it becomes the mode, all of which may have expressive meaning. The second is the linguistic mode which is usually combined with other forms, and in the process of message transmission, the linguistic mode and other modes usually work simultaneously, which is called constraint in meaning [21]. The last point is that users usually change existing patterns and create new ones in order to meet the needs of social messaging.

## 3. Application Design of Multimodal Assisted Scaffolded Instruction

### 3.1. Preinstructional Design Analysis

Before teachers carry out teaching activities, they should first clarify what the learning objectives of the course are and whether the teaching environment can meet the needs of the course and ensure that teaching activities can be carried out in an orderly manner. For subject teachers, the classroom objectives that are more important in instructional design are learning objectives, which are concrete and clear expressions of students' behavioral states after learning. In designing multimodal assisted scaffolding instruction, teachers should develop different levels of learning objectives according to different types of courses by analyzing the learning objectives. The objectives of the course and each chapter are identified, and the topics of learning are described in general terms at each level, including what students will learn; what practical skills they will acquire; what innovative work they will accomplish through research; and what learning potential they have.

**Figure 1:**
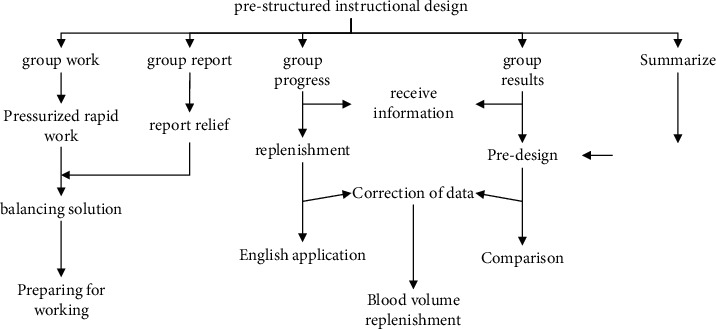
Prestructured instructional design work.

For the analysis of students' emotions and cognition, as shown in Figure 1, the starting point of teaching is judged, and the relevant knowledge and operational skills that students already have for learning a subject, as well as their attitudes toward the content of the cognitive subject, are understood. Second, the analysis should be based on the general characteristics of students' psychological, physical, and social environments in which they live. Teachers should identify which factors affect students' attitudes toward learning, including personality differences, age characteristics, and learning styles. Constructivist theory can be seen in many fields, and Hartman argues that it contains two thoughts: social constructivism and cognitive constructivism.

The pedagogical content should first aim at fulfilling the designed pedagogical objectives, which consist mainly of the ability of the students to acquire the competencies, knowledge, and technical skills needed to learn the subject as designed in the previous pedagogical design. The teaching content should be integrated with reality, classified in the form of its expression, distinguishing facts, theories, and understanding the difference between skills and techniques. The teacher should study the subject in relation to the real situation, conduct an in-depth study and analysis of the content of the textbook on the basis of ensuring the completion of the teaching of theoretical knowledge, and develop programs to develop students' ability to think independently and analyze problems. In the teaching process, the teacher should pay attention to communication with students, encourage communication between students while ensuring classroom discipline, and grasp students' abilities through communication; enhance trust between teachers and students; increase cutting-edge knowledge; and expand course knowledge and case studies. The use of modern teaching media devices has become an irreplaceable role in teaching activities.

### 3.2. Analysis of the Elements and Formation of Scaffolds in a Multimodal Teaching Environment

Again, by judging and analyzing students' psychological characteristics and learning styles, teachers can understand the way students receive information and the way students think and understand the different reactions of students to the external environment after the change. The traditional teaching environment provides scaffolding for students in the teaching process, mainly for teaching and learning, and does not meet other requirements for teaching and learning. The creation of a multimodal teaching environment takes into account various elements that can improve the efficiency of teaching and learning, in addition to meeting the scaffolding of the teaching and learning process.

**Figure 2:**
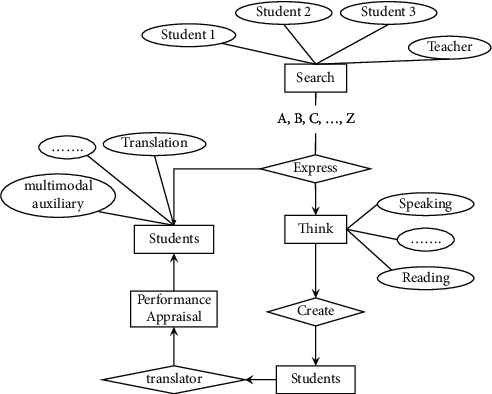
The construction of learning scaffolds for online learning activities.

1. Hardware. Scaffolding-assisted learning is provided in multimodal translating classrooms, primarily in terms of both groups and individuals. Group learning requires the provision of appropriate venues and facilities for groups, such as large classrooms with multimedia equipment capable of unified control, computers, and necessary supporting equipment. The use of large classrooms facilitates a lively classroom atmosphere and enhances activities between teachers and students as well as among students. Large classrooms can simulate interactive online teaching sessions for activities, where teachers use multimedia networks and with other teaching systems to scaffold students. Individual learning is to equip each student with a computer to which students can perform certain operations related to learning, and students give full play to the functions of the computer's network card, sound card and other hardware devices in the learning process. Teachers make appropriate adjustments to guide students in the learning process based on their judgment of the students, and adjustments should be flexible and varied according to the real situation in order to achieve the purpose of active student participation.
2. Software. It mainly includes online learning activities, computer software and teacher guidance. Online learning activities: mainly include browsing, searching, communicating, generating and evaluating, etc. The scaffolding provided is generally considered in terms of these aspects. Therefore the scaffolding provided for learners can be implemented by considering the relevant learning activity elements from these aspects above as well as learning resources and tools (as shown in Figure 2). The teaching environment mainly includes the hardware equipment and software configuration, and it is also necessary to clarify the basis of the students as the main teaching subjects, to analyze the students' existing abilities and levels before entering the classroom, and to grasp the students' attitudes toward the course through the analysis is also an important topic of this paper's research.

In this experiment, a total of 136 students from Classes 1, 2, 3 and 4 of Grade 16 were used as subjects, as shown in Table 1. The male to female ratio was basically the same in the four classes, and the average English grades were close to each other. Among these four classes, Class 1 and Class 4 were randomly selected to constitute the experimental group, with 69 students in the experimental group. Classes two and three served as the control group, with 67 students in the control group. This experiment was implemented in September 2021, and after one semester, the students completed the English courses they had taken. This experiment was made possible because the institute is a pilot college for the new curriculum reform at the university. During the experiment, all students followed the same English teacher in a comprehensive English course in a large multimedia classroom, and the textbook used was the Comprehensive English Course for University Experience, where they had four hours of English classes per week. The study participants had some common characteristics. For example, they had been learning English for approximately the same amount of time, had similar cultural and social backgrounds, and none had been to a native English-speaking country such as the United Kingdom, the United States, or Australia. The age of the study participants ranged from 18 to 24 years old. In terms of the English GCE scores of the study subjects, the students in the experimental group and the control group of the same major had an even distribution of GCE English scores. Before the experiment, the English language proficiency of the experimental subjects was basically at the same level.

**Figure 3:**
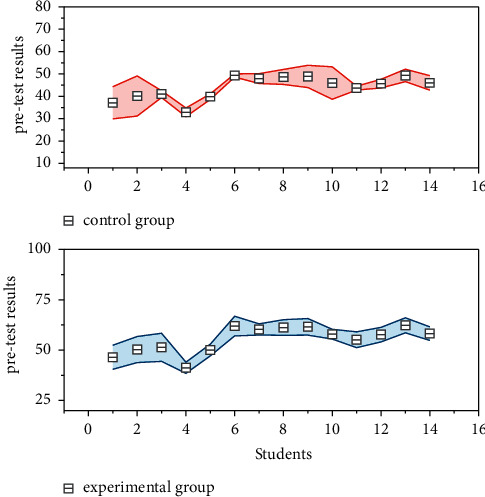
Pre-test analysis of the experimental and control classes.

The results of the experiment were analyzed by five main dimensions: the form of classroom interaction, learning motivation, learning autonomy, language skill use, and English performance. The English test allows for a comprehensive examination of students' English proficiency. The English tests before and after the experiment consisted of four parts: listening test, speaking test, reading test, and translating test, each with 25 points, totaling 100 points. In order to ensure that the difficulty levels of the pretest and post-test are comparable, the listening, reading, and translating test questions are selected from the English IV examinations for college students. For the test of students' speaking ability, since there is no quantitative and unified speaking scoring standard for the Level 4 exam for the time being, this experiment used the IELTS exam speaking scoring standard to rate students' speaking ability in four aspects: fluency and coherence, grammatical range and accuracy, vocabulary, and pronunciation. The scores of the control group and the experimental group before the experiment was conducted are shown in Figure 3, and the scores were analyzed by one-way ANOVA and independent samples *t*-test.

This experiment was conducted before and after the experiment for two groups of students in the English test, and the data collection and analysis of the pretest and post-test scores was completed, and the questionnaire was distributed after the experiment to test the operability of the experiment and analyze its application effect. By comparing the pretest English test scores of the two groups, the means of the two groups were 67.52 and 66.97, respectively, and the standard deviation scores were 10.399 and 9.364, with a small difference between the two groups and a significance of 0.746 > 0.05. The results of the one-way ANOVA and independent samples *t*-test both indicated that there was no difference in English scores between the two groups of students before the experiment. The process of the experiment was to implement different teaching programs for students in the experimental and control groups. In this study, questionnaires on teaching styles were administered to students in the experimental and control groups, respectively, after the experiment to reflect the differences between the experimental and control groups in the first dimension of the form of classroom interaction through students' subjective evaluations. Speaking practice activities and English video and audio materials were the items that took more time in the experimental group's classroom, and word-by-word analysis of articles was the least, which was in line with the group's interactive classroom teaching process; in the control group, grammar knowledge explanation and word-by-word analysis of articles were the items that took more time in the classroom, and speaking practice was the least, which was in line with the teacher-centered indoctrination teaching process.

### 3.3. Implementation of Multimodal Assisted Scaffolded Instruction

**Figure 4:**
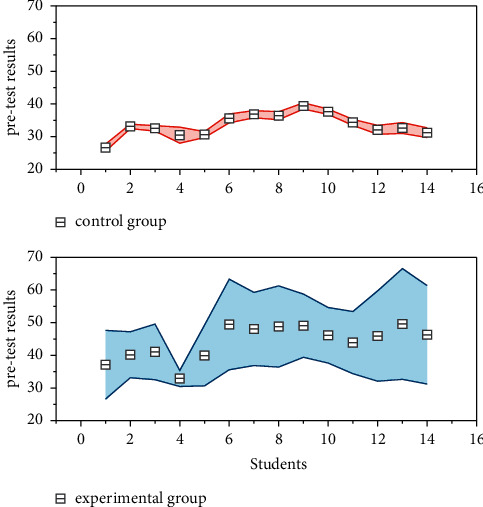
Post-test analysis of the experimental and control classes.

Moreover, the significance index is 0.926, which is higher than 0.05, which indicates that the English translating levels of the two classes are almost parallel, ensuring the validity of the post-test results (as shown in Figure 4). By analyzing the posttest results of the two classes, it was concluded that the mean score of the experimental class was 63.75 and the mean score of the control class was 60.11. The experimental class was almost 4 percentage points better than the control class, and this data indicated that the experimental class performed higher than the control class. The standard deviation of the experimental class was 13.83 and the standard deviation of the control class was 16, indicating that the gap between the translating scores of the students in the experimental class through practice was smaller compared to the control class. In addition, the significance index for the two classes was 0.011, which was lower than 0.05, implying that the difference between the two classes' scores was significant. In conclusion, the results indicate that there is a large difference in the performance of the two classes after the implementation of multimodal assisted scaffolding in the experimental class.

The average score of students in the experimental class was 63.75 in the post-test and 59.57 in the pretest. The post-test was higher than the pretest by more than 4 percentage points, and the average score of the two tests changed significantly; the significance index was 0, which indicated that there was a significant difference between the pre- and postscores of students in the experimental class. The smaller the standard deviation, the closer it is to the mean and smaller the gap between students' scores. The reduced standard deviation of the experimental class means that more scores are close to the mean and the gap between students is narrowed, which means that the experimental class students' scores have improved and the polarization is reduced. By analyzing the pretest scores of the two classes, it was found that the mean score of the experimental class was 59.57 and the mean score of the control class was 59.43; the standard deviation of the experimental class was 15.84 and the standard deviation of the control class was 16.10.This data analysis showed that the students' proficiency levels of the two classes tended to be the same.

## 4. Statistical Results and Analysis

This part is mainly based on the results of the two questionnaires. Since the two questionnaires were conducted before and after the experiment, comparing the results of the two questionnaires can show the main changes of students after they passed the experimental teaching, which mainly include their views on the importance of learning to write; their confidence in learning to write; their feedback on the translating teaching mode; and their ability of independent learning behavior, and their main changes include perceptions of the importance of learning to write; confidence in learning to write; and feedback on the translating teaching model, students' independent learning behavior ability and students' translating level. According to the analysis of the results of the former questionnaire, it can be seen that students generally have negative attitudes toward English translation, and most of them write with the attitude of making up the number of words, lacking good learning habits. Students feel uninteresting and tedious about the existing English translating teaching mode and have no confidence in translating good compositions. In contrast, the postexperimental questionnaire shows that students' attitudes have changed a lot, and they are very interested in the new translating classroom, which they think brings them a lot of fun and makes them really involved in translating; they can also learn a lot of English knowledge and skills. This comparative result means that students are more willing to accept the improved teaching method, and it also proves the value of the multimodal assisted scaffolding teaching model, which not only changes students' attitudes toward learning to write but also improves their translating skills and learning behaviors.

**Figure 5:**
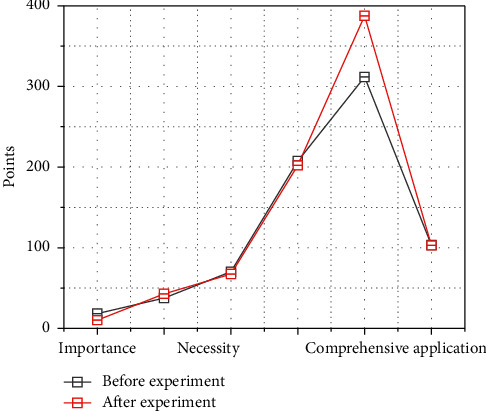
Comparison of the questionnaires before the experimental class and the control class.

Many students think that their low grades and poor English translating skills are related to the existing classroom teaching and are related to their own translating habits and translating methods. Most students do not have the habit of practicing translating on a regular basis and just cope with their assignments, and some of them even do serious translating only when they take exams. All the questions in the previous questionnaire were multiple-choice, and students selected the most appropriate answers according to the questions. A total of 126 questionnaires were distributed, 61 to the experimental class and 65 to the control class, with a 100% return rate. The questionnaires were mainly used to map the students' situations in the two classes and to investigate students' feedback on the existing classes, their attitudes and habits towards learning English translation and the factors affecting the teaching effectiveness. As shown in Figure 5, students' feedback on English translating learning was close to the same in both classes. Most students (88%) think that translating is important in English learning and should not be underestimated; they (73%) think it is necessary to learn to write well, and they also want to improve their translating skills. However, 70% of the students lost their confidence in their English translating and thought that translating was the shortcoming of learning English and that learning to translate was very difficult.

Only a few (20%) of the students usually keep an English diary to link their translating habits, and most (66%) of them have poor translating habits, they usually do not outline before translating, and 60% of the students mainly use Chinese English to write English, splicing multiple sentences into paragraphs without connecting words and transitions between sentences. Only 37% of the students could check and revise their compositions. Some students also thought that the teachers did not give them timely support and help when they encountered difficulties in translating, so it was difficult for them to improve their English translating skills.

Therefore, it is difficult to improve their English translation skills. At the same time, the survey found that 70% of the students were willing to use learning tools with pictures, audio, and video, and 60% of the students were willing to try to use online multimedia to learn translating and accept the new teaching mode brought by the information age. After the teaching experiment was completed, a questionnaire survey was conducted again for the students in the experimental class, and the content of the latter questionnaire survey was the same as the content of the former questionnaire. This questionnaire survey was designed to investigate the changes in students' interests and learning behaviors in learning English translation after the multimodal assisted scaffolding teaching, as well as students' feedback on the new teaching model. A total of 61 questionnaires were distributed, and the return rate was 100%.

**Figure 6:**
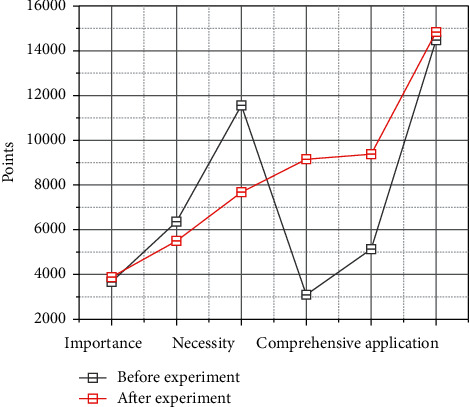
Comparison of questionnaires before and after the experimental class.

A comparative analysis of the two questionnaires before and after the experimental class (Figure 6) showed that the attitudes of the students in the class toward learning to translate had changed, and the number of students who thought translating was important for English learning had increased, but the number of students who thought a special English translating course should be offered did not change significantly. The number of students in the experimental class who were more interested in learning English translation than before the experiment increased by 16%, but the number of students who thought it was easier to learn English translation increased by only 8% due to the short duration of the experiment. Many students' confidence in learning English translating has increased, and the results of the questionnaire show that the number of students who think they are good writers has increased by 15%, and most students are confident in improving their translating skills through hard work, while the number of students who are not confident in improving their translating skills has decreased by 25%. In terms of students' motivation for translating, although the number of students who write for homework and exams has increased, this also shows that students no longer write aimlessly, but to do their homework better, and students begin to pay attention to translating and think that improving their translating performance is beneficial to improving the overall level of English learning.

**Figure 7:**
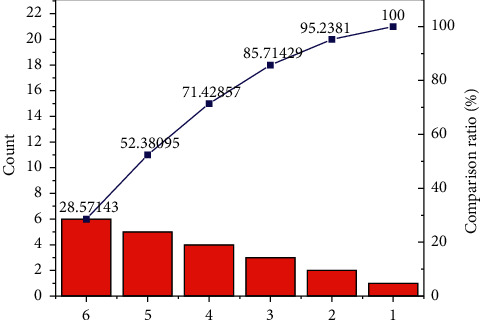
Comparison of the scaffolding approach and the traditional translating model.

The application of multimodal assisted scaffolding in college English translating teaching is a successful exploration of a new teaching model, which is different from the traditional English translating teaching model and takes students as the main body to create a colorful teaching form. The mean scores of these two classes are 59.57 and 59.43 respectively, which are almost the same, and the significance index is higher than 0.05, which means that the students of these two classes are at the same level (as shown in Figure 7). In the experimental group's training for output skills, the teacher conducted output skills training through group activities and task setting.

However, the mean scores of the two classes are 63.75 and 60.11 respectively, as shown in the post-test results of the experimental teaching. 63.75 and 60.11, respectively, and the significance index was 0.011, which was much lower than 0.05, indicating that after the experimental teaching, the experimental class and the control class showed a large difference in performance, and the experimental students made greater progress. However, if we look at the results of the pre and post-test of the experimental class alone, the data show that the performance of the post-test of the experimental teaching is higher than that of the pretest of the experimental teaching, and the significance index is 0. This result indicates that the performance of the experimental class has produced a change and has improved significantly compared with that of the pre-experimental; while the performance of the pre and post-test of the control class has not changed significantly and can be considered almost the same. Based on the above statements and the analysis of the data on the students' test scores, it can be concluded that the students' performance in the class of the multimodal assisted scaffolding teaching experiment improved significantly, the common problems in composition were improved, and the level of composition was improved. This is a side indication that the teaching model has been successful to some extent.

**Figure 8:**
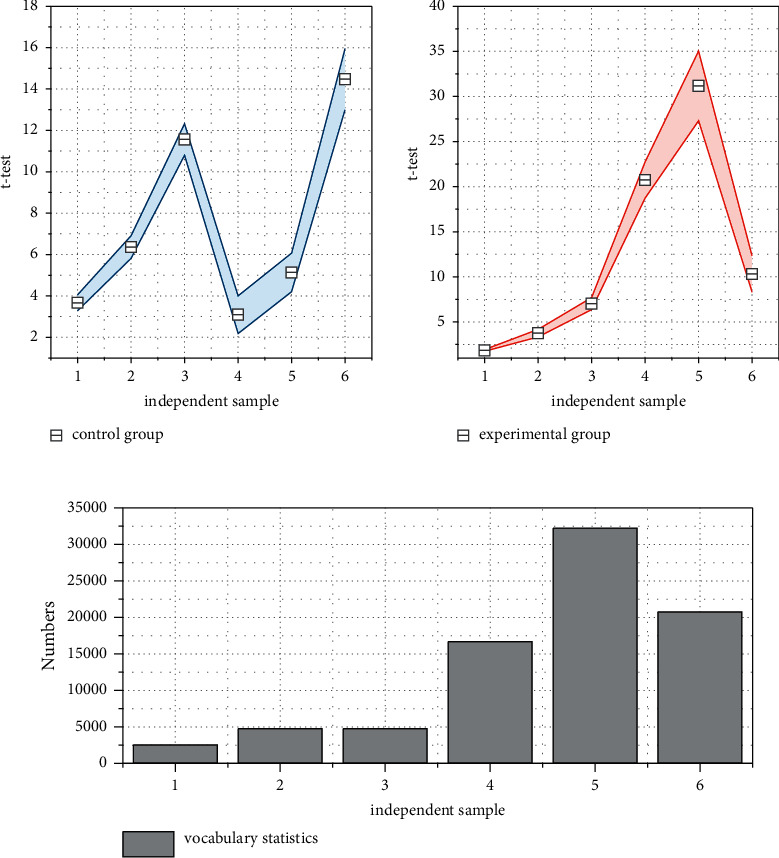
Results of an independent sample *t*-test for learning autonomy.

As shown in Figure 8, questions 11–17 examine students' autonomy in learning English. The higher the score of the questions, the greater the autonomy of the students. The mean values of questions 11–17 in the control group are smaller than those in the experimental group, and the *p* values are all less than 0.05, indicating that the difference between the two classes in terms of learning autonomy is significant, and the students in the experimental group are more autonomous in learning English, and hypothesis two is valid. In the open-ended practice for speaking, there is a difference in information between students in the group; only through communication in English can students get each other's information. For example, students A and B are given a form at the same time, but B does not have the information that A has on the form. Thus, B must have a conversation to get the information on A's form. Information differences can give students a purpose for communication and allow them to practice oral expression while completing group tasks. Use topics like debates, finding differences, speed dating, asking for directions, restaurant ordering, and other real-life scenarios that might be used in speaking practice. Students work in two-person or four-person groups before conducting a whole-class activity. During the group activities, the teacher supervises the class as a supervisor and facilitator, listening to the ideas presented by the students and not interfering in their discussions. If students ask for help with a problem they do not know, the teacher gives a little guidance and assistance to the students.

The ANOVA results showed that the mean square difference between the experimental and control groups was much larger than the mean square difference within the groups, and the differences in the 138 students' scores were mainly between groups. The significance level of the F-statistic was much smaller than 0.05, so it can be considered that the differences in the scores between the experimental and control groups were statistically significant. The mean scores of 65.69 and 75.30 for the control and experimental groups, respectively, were significantly higher in the class with interactive group teaching than in the control group, and a further *t*-test of the sample means also indicated that this difference was significant. This indicates that interactive group teaching is helpful in improving students' academic performance, and hypothesis four holds. Students in the control group were less motivated to learn English in the classroom. After 20 minutes of class, students in the control group generally shifted their attention. In the last fifteen minutes of class, students' patience was at its lowest and most of them had difficulty staying focused on the lesson. The level of participation in learning activities in the classroom was not high. After the teacher asked a question, only one or two students would respond to the teacher, and most students were silent. The results of the questionnaire showed that more than half of the students in the control group said that they wanted to answer after the teacher asked a question, but they were afraid to raise their hands, hoping that the teacher would call on them. In the control group's classroom, the teacher was the authority on knowledge, and students needed to be “careful with their words” for fear of making mistakes in class.

## 5. Conclusion

English learning occupies a very important position in college students' learning, and English translating is a skill that must be mastered to learn English, and translating can be used to measure the comprehensive level of college students' English learning. However, surveys show that English translation is still a difficult part of the English learning process for college students. Traditional translation teaching only focuses on the result and ignores the process; the classroom atmosphere is dull; students are not highly motivated to write; and students' knowledge of translating and its skills are lacking. Therefore, translating teaching should combine results and process and explore teaching methods that can improve students' interest and translating ability. In this paper, scaffolded teaching is mainly applied to college English translating teaching, and multimodal teaching assists scaffolded teaching. At the same time, the group interaction cultivated the students' spirits of cooperation and exploration, which will contribute to their long-term development in the future. In contrast, students in the control group behaved as passive receivers in the classroom, believing that learning needed to be carried out with the participation of the teacher. Since the teaching schedule and content were strictly controlled by the teacher, students in the control group lacked the ability to plan themselves and learn on their own, and when they encountered problems, students in the control group relied excessively on the teacher to explain. The results showed that students in the experimental group mastered language skills significantly better than those in the control group. When faced with difficult input materials, the experimental group students were proficient in using techniques such as keyword targeting, topic prediction, and grammatical structure-assisted guessing to analyze and process the information. Students in the control group experienced anxiety when faced with difficult input materials and had to translate with the help of a toolkit to complete the task.

In the future, questionnaires on teaching styles will be administered to students in the experimental and control groups, respectively, after the experiment to reflect the differences between the experimental and control groups in the first dimension of the form of classroom interaction through students' subjective evaluations.

## Figures and Tables

**Table 1:** List of teaching objects.

Experimental classes	Number of people	Academic performance
1	23	69 ± 11.5
2	24	72 ± 12.4
3	43	62 ± 15.9
4	46	81 ± 14.9

## Data Availability

The data used to support the findings of this study are available from the corresponding author upon request.
